# CAREER SELF-MANAGEMENT OF LATE LIFE WORK

**DOI:** 10.1093/geroni/igad104.1105

**Published:** 2023-12-21

**Authors:** Harvey Sterns

**Affiliations:** The University of Akron, Akron, Ohio, United States

## Abstract

The choice to retire can be gradual or sudden depending on the planning, the opportunities presented, and crises encountered. Issues of self-management will be examined focusing on the challenges faced by older faculty members seeking to continue working. Present and future older academics are increasingly pressed to take active involvement and responsibility for career management. A multidimensional model for self-management of career and retirement will be presented.

